# CCNE1 Exerts a Protective Effect on Parkinson's Disease by Regulating Ferroptosis‐Related Proteins

**DOI:** 10.1002/brb3.71110

**Published:** 2025-11-29

**Authors:** Jia Fu, Jing Zhao, Na Mi, Chao Zhang, Yali Zhang, Lifen Yao

**Affiliations:** ^1^ Chifeng Municipal Hospital Chifeng City Inner Mongolia Autonomous Region China; ^2^ The First Affiliated Hospital of Harbin Medical University Harbin Heilongjiang Province China

**Keywords:** Parkinson's disease, ferroptosis, CCNE1, Mendelian randomization

## Abstract

**Background:**

The pathogenesis of Parkinson's disease (PD) is closely linked to ferroptosis, yet the mechanism by which ferroptosis‐related proteins modulate PD risk through genetic variations remains unclear.

**Methods:**

We performed two‐sample Mendelian randomization (MR) analyses using protein quantitative trait loci (pQTLs) from the UKB‐PPP and deCODE studies as instrumental variables, combined with PD genome‐wide association study (GWAS) data. The inverse variance weighting (IVW) method served as the primary analytical approach to identify PD‐associated proteins. To assess the role of ferroptosis‐related proteins in PD, a two‐step MR mediation analysis was conducted, followed by multiple sensitivity analyses. Transcriptomic datasets were analyzed to confirm the differential expression of genes encoding prioritized proteins. Publicly available single‐cell RNA sequencing data were utilized to investigate cell‐type‐specific expression patterns and functional pathways of key proteins.

**Results:**

A total of 210 proteins significantly associated with PD were identified (*p*<0.05). Mediation analysis demonstrated that CCNE1 mediated 44.17% of the neuroprotective effect (*β* = −0.503, *p* = 5.8 × 10^−4^) through upregulating PARP16 expression (*β* = 0.318, *p* = 2.36 × 10^−18^), with CCNE1's differential expression further validated in transcriptomic datasets. Single‐cell analysis demonstrated that CCNE1 and PARP16 are specifically highly expressed in neurons (*p* < 0.05), with neurons being significantly enriched in neural survival and synaptic plasticity pathways. The cell interaction network revealed that neurons specifically communicate with astrocytes via the NRG3‐ERBB4 pathway.

**Conclusion:**

This study provides the first molecular insight into how CCNE1 exerts neuroprotective effects through the regulation of the ferroptosis key protein PARP16, offering a novel perspective for PD mechanism research.

## Graphical abstract

This study provides the first molecular insight into how CCNE1 exerts neuroprotective effects through the regulation of the ferroptosis key protein PARP16, offering a novel perspective for Parkinson's disease mechanism research.

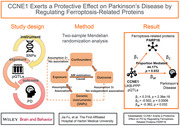

## 1 Introduction

Parkinson's disease (PD), the second most prevalent neurodegenerative disease globally, affects almost 10 million individuals, and its incidence continues to rise with population aging, placing a substantial socioeconomic burden (Rodríguez‐Molinero 2025; Zhu et al. 2024). Currently, the main treatment for PD is dopamine replacement therapy, which cannot halt the progressive loss of dopaminergic neurons. Therefore, research into its pathological mechanisms and the development of new therapeutic targets are urgently needed (Adam et al. 2023; Alotaibi et al. 2024). Breakthrough advances in proteomics have offered new insights into PD‐related molecular events, especially the pathological aggregation of α‐synuclein and the mechanisms behind brain iron homeostasis disruption (Khan 2025; Lumpkin et al. 2024; Toomey et al. 2022; Yan et al. 2024; Yao et al. 2024). Recent evidence indicates that ferroptosis, a type of programmed cell death driven by iron‐dependent lipid peroxidation, could be a key convergence point for the multiple pathological features of PD (Awasthi et al. 2025). Autopsy studies have revealed characteristic iron deposition in the substantia nigra of PD patients and a significant reduction in glutathione peroxidase 4 (GPX4) activity, both of which strongly correspond to the core features of ferroptosis (Lin et al. 2022; Yao et al. 2024). Despite multiple studies confirming that ferroptosis contributes to dopaminergic neuron death (Z. Lin et al. 2025; Zhou et al. 2024), the molecular regulatory network and its causal link to PD pathogenesis are still unclear.

Currently, PD proteomics research predominantly remains confined to single‐omics approaches, which inadequately control for confounding factors, while conventional observational studies are inherently limited in resolving reverse causation and similar methodological challenges (Huang et al. 2023; Sallis et al. 2023). Mendelian randomization (MR), a robust causal inference methodology in epidemiological research, leverages genetic variants (single nucleotide polymorphisms [SNPs]) as instrumental variables to infer exposure‐outcome relationships, thereby circumventing confounding biases and reverse causation (Emdin et al. 2017; Larsson et al. 2023; Song et al. 2023). This approach has been successfully implemented in recent years for both mechanistic elucidation and molecular target identification across multiple disease systems (Huang et al. 2023; Larsson et al. 2023; S. C. Lin et al. 2025; Sallis et al. 2023; Song et al. 2023; Wang et al. 2024; Zhang et al. 2022). Building upon this framework, Mendelian mediation analysis (MR‐mediation) dissects the causal pathway from exposure (X) → mediator (M) → outcome (Y), enabling quantification of mediation proportion and providing genetic‐level evidence for the mechanistic roles of biomarkers in disease pathogenesis(S. C. Lin et al. 2025; Wang et al. 2024; Zhang et al. 2022). Our study pioneers the integration of protein quantitative trait loci (pQTL) with multidimensional ferroptomics data to systematically identify PD‐associated ferroptosis regulators and reconstruct their interactomes using MR‐based frameworks, ultimately contributing to the discovery of genetically validated therapeutic targets for PD.

## 2 Materials and Methods

### 2.1 Study Design

This study adopts a two‐stage analytical framework to systematically identify potential pathogenic/protective proteins in PD and elucidate their underlying mechanisms. First, PD‐associated proteins were screened using UK Biobank‐Protein Phenotype Project (UKB‐PPP) pQTL data (Emdin et al. 2017), followed by mediation analysis of ferroptosis‐related proteins to uncover their potential pathogenic pathways. All analyses rigorously adhered to the three core assumptions of MR (strong instrument‐exposure association, independence assumption, and exclusion restriction) (18) and complied with the STROBE guidelines for observational research (Skrivankova et al. 2021).

### 2.2 Data Sources and Preprocessing

We extracted pQTL data from 2940 European‐ancestry participants in the UKB‐PPP database (B. B. Sun et al. 2023). After stringent quality control (SNP selection *p* < 5×10^−8^, LD *r* ^2^< 0.1 within 10,000 kb windows), 2619 independent pQTLs were retained for initial PD protein screening. Ferroptosis‐associated pQTLs were derived from 4907 Icelandic individuals in the deCODE database (Ferkingstad et al. 2021). Following identical quality control (QC) steps, 4759 high‐confidence pQTLs were overlapped with ferroptosis driver/suppressor genes curated in FerrDb to construct the final dataset. PD GWAS summary statistics were obtained from the IPDGC meta‐analysis (Nalls et al. 2019), comprising 33,674 cases and 449,056 controls (total *N* = 482,730). The transcriptomic data utilized in this study were all obtained from the Gene Expression Omnibus (GEO; https://www.ncbi.nlm.nih.gov/geo/), specifically including the following datasets: bulk RNA sequencing data of substantia nigra tissues from PD patients and controls (GSE49036, GSE20292), midbrain single‐cell RNA sequencing data comprising both PD and control samples (GSE157783), and single‐cell transcriptomic data of the substantia nigra also including PD and control samples (GSE178265).

### 2.3 MR Analysis

Two‐sample MR analysis was conducted using the TwoSampleMR package (v0.6.6), primarily employing the inverse variance weighting (IVW) method to estimate causal effects, while weak instrumental variables with *F*‐statistics <10 were excluded. During the preliminary screening and mechanistic exploration phases, conventional significance thresholds (*p* < 0.05) were employed for subsequent analyses, including MR and mediation effects, to prevent excessive reduction in statistical power and loss of potential biological significance associated with multiple testing correction (L. Wang et al. 2023). Mediation effect analysis was performed using a two‐step MR approach to assess the mechanism by which PD‐related proteins act through the ferroptosis pathway. Specifically, the total effect of the candidate protein pQTL → PD (*β*_all_) was first calculated, followed by the evaluation of the effects of the candidate protein pQTL → ferroptosis‐related proteins (*β*_1_) and ferroptosis‐related proteins → PD (*β*_2_). The mediation effect (*β*_1_×*β*_2_) and mediation proportion ([*β*_1_×*β*_2_]/*β*_all_×100%) were then calculated, with a selection criterion of *p *< 0.05, mediation proportion >10%, and consistent effect direction. Expression validation analysis of MR‐identified PD‐related protein‐coding genes was conducted: publicly available transcriptomic mRNA expression data from the GEO database were analyzed using R (v4.3.0), with the Wilcoxon rank‐sum test (*p* < 0.05) used to compare expression differences between the control and PD groups, and results were visualized using the ggplot2 package.

### 2.4 Sensitivity Analyses

To ensure the robustness of our findings, we conducted comprehensive sensitivity analyses. Heterogeneity across instrumental variables was quantified using Cochran's Q statistic (*p* < 0.05 indicating significant heterogeneity). Horizontal pleiotropy was evaluated via MR‐Egger regression, with significant pleiotropy defined by an intercept *p* ≤ 0.05. Outlier detection and correction were performed using MR‐PRESSO, where a global test *p* > 0.05 was considered acceptable. Finally, leave‐one‐out analysis was conducted to confirm the stability of the effect estimates.

### 2.5 Single‐Cell Transcriptomic Analysis

Single‐cell transcriptomic analysis was carried out using the Seurat package (v5.1.0; Satija, Farrell, Gennert, Schier, and Regev 2015). First, QC was performed on the raw data by eliminating cells with fewer than 200 expressed genes and genes expressed by fewer than three cells, while excluding cells with abnormal gene counts (≤200 or ≥6000) and UMI counts (≤500 or ≥20,000). The data were normalized using the NormalizeData function, followed by the selection of 2000 highly variable genes using the FindVariableFeatures function. Principal component analysis (PCA) was conducted, with significant principal components identified using the ElbowPlot. Batch effects were corrected using the harmony package (v1.2.0; Korsunsky et al. 2019), and UMAP dimensionality reduction and clustering were performed at a resolution of 0.3. Cell types were manually annotated based on published marker genes (Quan et al. 2024; Smajić et al. 2022), focusing on the analysis of cell‐specific expression patterns of key characteristic genes, and selecting subpopulations of cells with high expression of target biomarkers. Functional pathway enrichment analysis was subsequently conducted using ReactomeGSA (https://github.com/reactome/ReactomeGSA), followed by systematic reconstruction of intercellular communication networks via CellChat (v1.6.1) (Fang et al. 2022).

### 2.6 Statistical Analysis

All statistical analyses in this study were performed using the open‐source R software (version 4.3.0; http://www.R‐project.org). A *p*‐value < 0.05 was considered statistically significant.

## 3 Results

### 3.1 Identification and Expression Validation of PD‐Associated Protein‐Coding Genes

**FIGURE 1:**
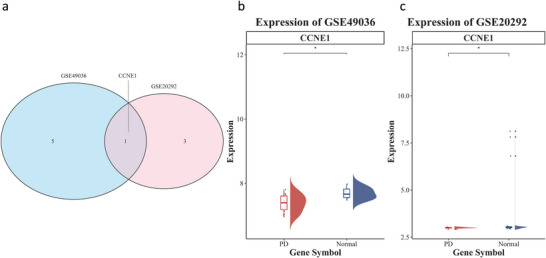
Differential expression analysis of transcriptome data. (a) Venn diagram of candidate genes. (b) Expression of CCNE1 in GSE20292. (c) Expression of CCNE1 in GSE49036.

This study utilized UKB‐PPP pQTL data as exposure variables and PD as the outcome variable. Through a two‐sample MR framework, the IVW method identified 210 proteins demonstrating significant causal associations with PD (*p* < 0.05; Table S1). To further investigate the transcriptomic regulatory characteristics of genes encoding these proteins, we conducted differential expression analysis on two independent human brain substantia nigra transcriptomic datasets (GSE49036 and GSE20292). The GSE49036 dataset consists of eight normal controls and 15 PD cases with Braak α‐synuclein stage ≥3, while the GSE20292 dataset includes 11 normal controls and 18 PD cases. The results of the Wilcoxon rank‐sum test revealed significant downregulation of the CCNE1 gene expression in PD patients (*p* < 0.05) (Figure 1a–c), which is consistent with the negative causal effect (*β* < 0) observed in the MR analysis (Table S1).

### 3.2 Identification of Ferroptosis‐Related Proteins and MR Analysis of Their Association with PD

**FIGURE 2:**
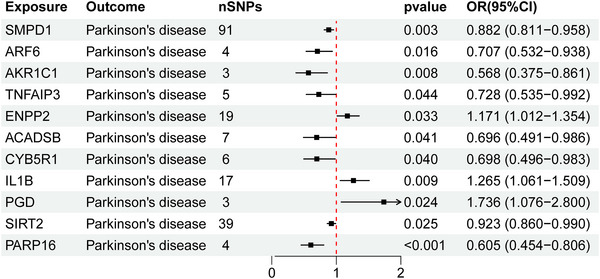
Causal effects of ferroptosis‐related proteins on PD risk assessed by inverse‐variance weighted (IVW) regression.

We systematically integrated 483 ferroptosis regulators from FerrDb with 4,907 protein‐coding genes in the Icelandic pQTL database, which led to the identification of 159 ferroptosis‐associated genes with valid pQTLs through intersection analysis. After accounting for protein isoforms, the final dataset comprised 165 ferroptosis‐related proteins, which cover core pathway components and their genetic variants. Using two‐sample MR with ferroptosis protein pQTLs as instruments and PD GWAS as outcomes, we estimated causal effects via IVW. MR analyses identified 11 ferroptosis‐related proteins significantly causally associated with PD risk (Figure 2). Of these, eight proteins (SMPD1, ARF6, AKR1C1, TNFAIP3, ACADSB, CYB5R1, SIRT2, and PARP16) showed protective effects (*β* < 0, *p* < 0.05), while ENPP2, IL1B, and PGD exhibited risk‐enhancing effects (*β* > 0, *p* < 0.05).

### 3.3 The Mechanism by Which CCNE1 Mediates PD Pathogenesis Through Ferroptosis‐Related Proteins

To investigate whether CCNE1 influences PD risk through the regulation of the ferroptosis pathway, we conducted a two‐step MR mediation effect analysis. The results showed that the total effect of CCNE1 on PD (*β*_all_ = −0.362, *p* = 0.032) is mediated by three key ferroptosis‐related proteins—ADP‐ribosylation factor 6 (ARF6), cytochrome b5 reductase 1 (CYB5R1), and Poly (ADP‐ribose) polymerase family member 16 (PARP16), with the mediating effect of PARP16 being the strongest (mediated proportion 44.17%). Specifically, CCNE1 significantly upregulates the expression of PARP16 (*β*1 = 0.318, *p* = 2.36 × 10^−18^), while PARP16 itself shows a significant negative correlation with PD risk (*β*2 = −0.503, *p* = 5.8 × 10^−4^), which reveals the potential molecular pathway of CCNE1‐PARP16‐PD. The mediation effects and specific parameters of the remaining two mediating proteins (ARF6 and CYB5R1) are provided in Table 1. Reverse MR analysis indicated no significant causal effects of PD on the aforementioned protein levels (Table S2), effectively ruling out reverse causality due to disease progression‐induced molecular phenotypic changes and further supporting the reliability of the causal direction identified in the original mediation analysis. These results systematically elucidate for the first time the molecular mechanism by which CCNE1 influences PD risk through the regulation of key ferroptosis proteins.

**TABLE 1:** Mediation analysis of ferroptosis‐related proteins, CCNE1, and PD risk.

Ferroptosis‐related proteins	PD‐related proteins	*β*_all_	*β*_1_	*β*_2_	Indirect effect *β*	Direct effect *β*	*p*‐value	Proportion mediated (%)
ARF6	CCNE1	−0.362	0.351	−0.347	−0.122	−0.240	0.019	33.68%
CYB5R1	CCNE1	−0.362	0.390	−0.359	−0.140	−0.221	0.032	38.74%
PARP16	CCNE1	−0.362	0.318	−0.502	−0.160	−0.202	0.032	44.17%

### 3.4 Sensitivity Analysis

This study uses multidimensional sensitivity analysis to validate the reliability of MR results. To assess the heterogeneity of instrumental variables, we employed Cochran's Q test, MR‐Egger regression, and MR‐PRESSO global test. In the pleiotropy test, no significant pleiotropy was found, whereas Cochran's *Q* test revealed slight heterogeneity (see Tables S3–S6). Despite the relatively minor impact of heterogeneity on the overall results, we optimized the analysis using the IVW random‐effects model for SNPs with significant heterogeneity (Q test *p* < 0.05) and the IVW fixed‐effects model for SNPs showing no significant heterogeneity. MR‐PRESSO did not identify any significant outlier SNPs, and the leave‐one‐out analysis confirmed the stability of causal effect estimates, further reinforcing the robustness of the findings.

### 3.5 Single‐Cell Transcriptomic Analysis

**FIGURE 3:**
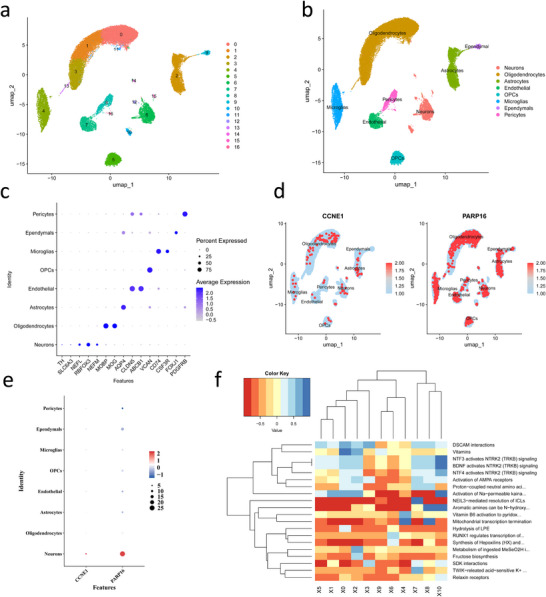
Cellular clustering analysis and distribution of key protein‐coding genes across cell clusters. (a) UMAP visualization of integrated sample clustering. (b) Bubble plot illustrating marker gene expression patterns. (c) UMAP projection of cellular clusters. (d) UMAP mapping of key protein‐coding gene expression distribution. (e) Bubble plot highlighting key protein‐coding gene expression across distinct cellular clusters. (f) Heatmap depicting functional enrichment of pivotal cellular processes.

**FIGURE 4:**
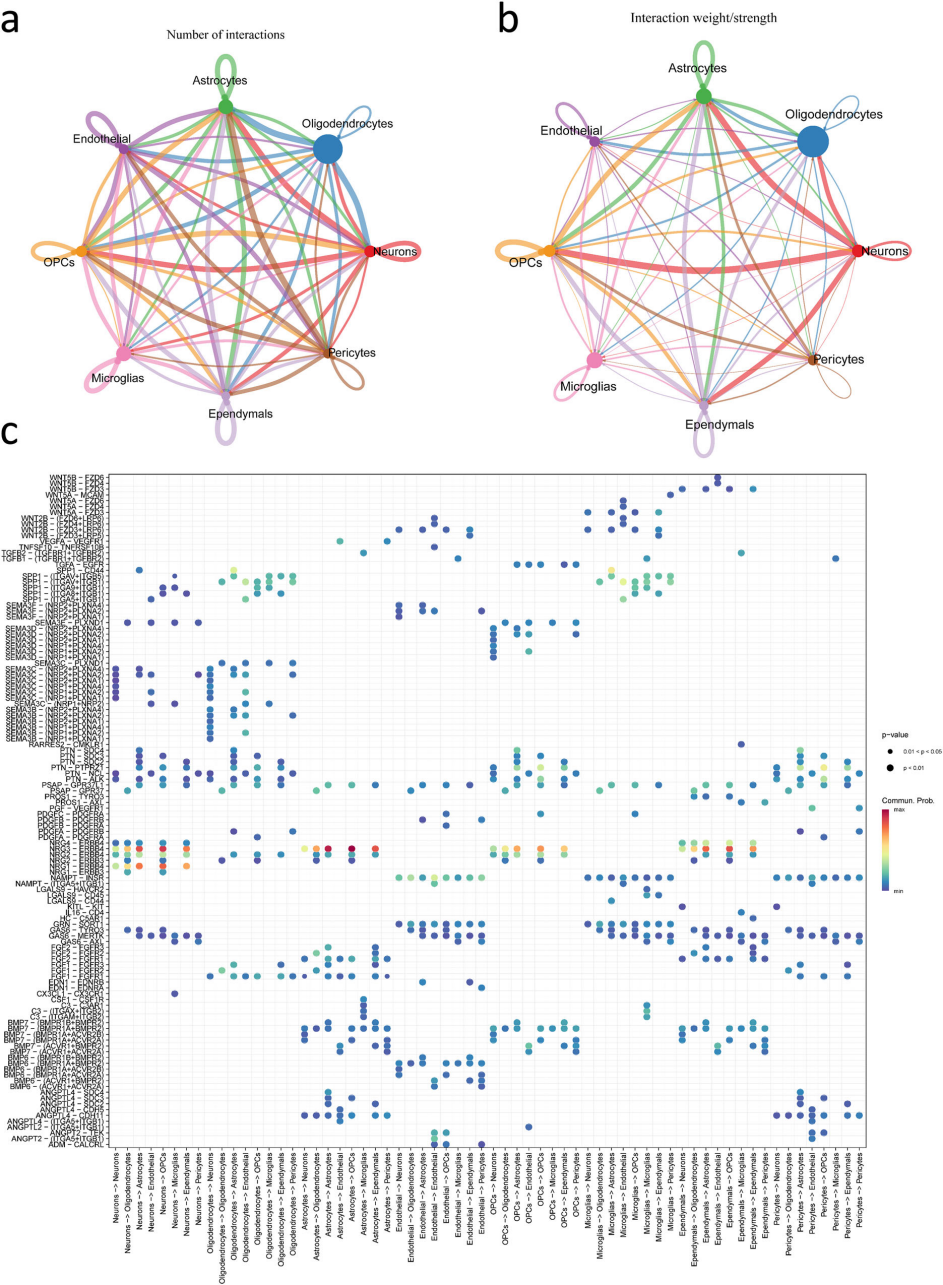
Cell–cell communication network analysis. (a) Peripheral circle dimensions correlate with cellular abundance across clusters. (b) Interaction probability heatmap depicting signaling intensity. (c) Receptor‐ligand interaction network visualized via proportional bubble plots.

The outcomes of the single‐cell dataset quality control before and after filtering are presented in Figure S1a,b. Cell clustering was performed based on 20 principal components (PCs) (Figure S1c), and after UMAP dimensionality reduction, 17 cell clusters were identified (Figure 4a). These clusters were annotated into eight distinct cell types (Figure 3b,c) according to marker genes, including neurons, oligodendrocytes, microglia, astrocytes, oligodendrocyte precursor cells (OPCs), pericytes, endothelials, and ependymals. Notably, gene expression feature analysis revealed that CCNE1 and PARP16 show a specific high‐expression pattern in the neuronal population (Figure 3d,e), indicating that these genes may have important roles in neuronal biological functions. Further functional enrichment analysis revealed that the neuronal population was significantly enriched in several signaling pathways closely associated with nervous system function (Figure 3f), such as “DSCAM interactions”, “Vitamins metabolism”, “NTF3 activates NTRK2 (TRKB) signaling”, “BDNF activates NTRK2 (TRKB) signaling”, and “NTF4 activates NTRK2 (TRKB) signaling”. These pathways are known to have crucial neuroprotective roles in regulating neuronal survival, maintaining synaptic plasticity, and resisting oxidative stress. To investigate the potential pathogenic mechanisms of PD, we performed a comparative analysis of the cell communication networks between different cell types in the PD group and normal samples. The analysis revealed that in PD samples, a significant interaction between neurons and astrocytes was established through the NRG3‐ERBB4 ligand–receptor pair (Figure 4a–c). This discovery indicates that NRG3–ERBB4 interactions could play a critical role in PD progression.

To validate the universality of our findings in a core region of PD, we performed an independent analysis using single‐cell RNA sequencing data from human substantia nigra pars compacta (SNpc; dataset GSE178265). Cell clustering was performed based on the top 20 principal components, yielding 18 distinct clusters after UMAP dimensionality reduction (Figure S2a). These clusters were annotated into eight major cell types, including dopaminergic neurons, using established marker gene expression profiles (Figure S2b,c). Signature gene expression analysis revealed that both CCNE1 and PARP16 were specifically elevated in dopaminergic neurons (Figure S2d,e). Functional enrichment analysis further demonstrated that dopaminergic neurons were significantly enriched in pathways related to “NTF3 activates NTRK2 (TRKB) signaling”, “BDNF activates NTRK2 (TRKB) signaling”, and “Presynaptic depolarization and calcium channel opening” (Figure 2f), which are critically involved in neuronal survival, synaptic plasticity, and neuroprotection. Cell communication analysis again revealed markedly enhanced neuron–astrocyte interactions mediated by the NRG3–ERBB4 ligand–receptor pair in the SNpc of PD patients (Figures S3 and S4), consistent with our observations in the midbrain dataset.

## 4 Discussion

This study, for the first time, demonstrates the protective role of CCNE1 in reducing PD risk through the regulation of ferroptosis‐related proteins by integrating multiple omics data. By integrating two‐sample MR, mediation analysis, and transcriptomic data, we confirm that genetically determined high expression of CCNE1 significantly lowers PD risk (*β* = −0.362, *p* = 0.032), with this effect primarily mediated by PARP16 (mediated proportion: 44.17%). This discovery not only provides novel causal evidence for the molecular mechanisms underlying PD but also highlights the unique strengths of the MR method in dissecting complex disease multi‐omics networks.

CCNE1, a crucial regulator of the G1/S phase transition in the cell cycle, has predominantly been studied in the context of cancer (Au‐Yeung, Mileshkin, and Bowtell 2023; Fu et al. 2023; Gorski et al. 2020). However, this study shows that its protective effect in PD might occur independently of classical cell cycle regulation. In contrast, while the abnormal activation of Cyclin D1/E2 in the MPTP‐induced PD model can worsen neuronal damage (X. Zhang et al. 2023), MR analysis indicates that lifelong high expression of CCNE1 is strongly linked to a reduced risk of PD. This contradiction might stem from disease stage‐specific regulation—during the pathological process of PD, compensatory downregulation of CCNE1 could suppress abnormal cell cycle reactivation. Meanwhile, genetically determined lifelong high expression confers neuroprotection through nonclassical mechanisms, such as the regulation of ferroptosis. Mechanistic analysis notably reveals that the protective effects of CCNE1 are significantly associated with ferroptosis‐related proteins such as ARF6, CYB5R1, and PARP16. The mediating role of PARP16 stands out the most, suggesting it could be a key link between CCNE1 and the ferroptosis pathway.

Ferroptosis, a form of iron‐dependent lipid peroxidation‐driven cell death, exhibits pathological features (iron accumulation, reactive oxygen species accumulation, and lipid peroxidation) that closely resemble the degenerative damage observed in dopaminergic neurons in PD (Agostini et al. 2023; Awasthi et al. 2025; Zhou et al. 2024). Recent research indicates that targeting and inhibiting ferroptosis holds promise in slowing the progression of PD and may serve as a potential therapeutic strategy(Fei and Ding 2024; Jiao et al. 2025; Y. Wang et al. 2023; Zhou et al. 2024). This study found that ferroptosis‐related proteins (ARF6, CYB5R1, PARP16) significantly mediate the relationship between CCNE1 and PD risk, suggesting that CCNE1 may play a crucial role in PD pathogenesis by regulating the ferroptosis pathway. Previous studies have demonstrated that ARF6 regulates the ferroptosis process through lipid peroxidation and iron metabolism (Geng and Wu 2022), and CYB5R1, as a key regulator of redox homeostasis, controls lipid peroxidation through H_2_O_2_ generation (Hall et al. 2022; Urano et al. 2025). It is worth noting that PARP16 showed the highest mediation proportion (44.17%) in the mediation effect model of this study, indicating its pivotal role in the CCNE1‐PD signaling axis. The PARP family is extensively involved in DNA damage repair, maintenance of genomic stability, and regulation of cell death (Alborzinia and Friedmann Angeli 2024; Lei et al. 2024). It participates in several cellular processes, including chromatin remodeling, transcriptional regulation, and energy metabolism, by catalyzing the poly (ADP‐ribosyl)ation (PARylation) of substrates (Pinton et al. 2022; X. Sun et al. 2023; Tan et al. 2023; Tolić et al. 2022; Vainonen et al. 2023). Research has confirmed that members of the PARP family (such as PARP1) play potential protective roles by regulating ferroptosis (Bondar and Karpichev 2024; Lei et al. 2024). For instance, in BRCA1‐deficient tumors, PARP inhibitors promote lipid peroxidation accumulation by downregulating GPX4 expression, thereby triggering ferroptosis (Lei et al. 2024). PARP1 reduces oxidative stress levels by repairing DNA damage, indirectly inhibiting the occurrence of ferroptosis (Bondar and Karpichev 2024). The expression of ferroptosis‐related proteins, such as SLC7A11, may be regulated by the PARP family, thus affecting their antiferroptosis function (Alborzinia and Friedmann Angeli 2024). These findings offer a theoretical basis for the CCNE1→PARP16→ferroptosis→PD regulatory axis. Further single‐cell transcriptomic analysis showed that CCNE1 and PARP16 are specifically highly expressed in dopaminergic neurons, which are significantly enriched in the “DSCAM interactions”, “NTF3/BDNF/NTF4‐NTRK2 signaling activation”, and “Vitamins” pathways, suggesting that the CCNE1‐PARP16 axis may combat ferroptosis by maintaining neuronal homeostasis.

Beyond the endogenous regulatory mechanisms within neurons, this study also identified a new interaction pattern between neurons and astrocytes in the pathological context of PD. In the PD model, neurons establish a specific communication network with astrocytes through the NRG3‐ERBB4 ligand–receptor interaction. Previous research indicates that activation of the ErbB4 receptor suppresses d‐galactose‐induced neuronal ferroptosis and aging phenotypes (Dao et al. 2025). This offers a new perspective for elucidating the role of glial cell‐neuron interactions in PD. The above findings provide a systematic revelation of the multilayered regulatory mechanisms of the CCNE1‐PARP16 axis across spatiotemporal dimensions—at the cellular level, it maintains the survival of dopaminergic neurons via the ferroptosis pathway, and at the tissue level, coordinates neuroglial interactions via the NRG3‐ERBB4 signaling pathway, thus creating a synergistic protective network targeting PD pathology.

In conclusion, this study uncovers the neuroprotective mechanism of the CCNE1‐PARP16 regulatory axis in PD through a multidimensional evidence chain, offering potential biomarkers for the early diagnosis of PD, such as CCNE1 expression levels and PARP16 activity. It also establishes the theoretical basis for developing novel therapeutic strategies targeting the ferroptosis pathway. Further research should focus on identifying the specific molecular targets of PARP16 in ferroptosis and verifying the intervention effects of the CCNE1‐PARP16 regulatory axis in in vivo models.

This study has several limitations: first, although the MR analysis has been validated through multidimensional sensitivity analysis, its specific molecular mechanisms remain to be experimentally verified. Notably, this study lacks proteomic validation of the implicated PD‐related proteins. Future work will involve collecting cerebrospinal fluid samples from PD patients and employing proteomics technologies to quantitatively analyze these target proteins, thereby seeking to validate our genetic findings at the protein level. Second, the screening of ferroptosis‐related proteins is constrained by the annotation coverage of the FerrDb database, which may result in the omission of key regulatory nodes, thus compromising the integrity of the pathway network. Third, the instrumental variables exhibit some heterogeneity, and although this was corrected using a random‐effects model, it still might reflect unaccounted‐for biological confounding pathways. Lastly, this study only involved a cohort of European descent, so future research should be conducted to further assess the generalizability of the results across diverse racial backgrounds.

## 5 Conclusion

This study, by integrating MR analysis, mediation analysis, public transcriptomic data, and single‐cell transcriptomic data, systematically reveals the causal mechanism by which CCNE1 reduces PD risk through the genetically driven upregulation of PARP16 expression, inhibiting the ferroptosis pathway. This finding not only extends the understanding of the nonclassical functions of cyclins in neurodegenerative diseases but also provides a crucial molecular foundation for the development of neuroprotective therapies based on ferroptosis regulation.

## Author Contributions

**Jia Fu**: conceptualization, software, formal analysis, methodology, writing – original draft. **Jing Zhao**: conceptualization, software, formal analysis, methodology, writing – original draft. **Na Mi**: investigation, formal analysis, data curation, visualization. **Chao Zhang**: investigation, data curation, validation. **Lifen Yao**: conceptualization, funding acquisition, supervision, resources, writing – review and editing. **Yali Zhang**: conceptualization, project administration, supervision, resources, writing – review and editing.

## Funding

This work was supported by the Science and Technology Program of the Joint Fund of Scientific Research for the Public Hospitals of Inner Mongolia Academy of Medical Sciences (no. 2024GLLH0988, 2023GLLH0312), the National Natural Science Foundation of China (no. 82471259), and the Heilongjiang Provincial Natural Science Foundation (no. ZD2023H004).

## Conflicts of Interest

The authors declare no conflicts of interest.

## Ethics Statement

The present study used only summary‐level statistics from publicly available datasets, and no additional ethics approval was required.

## Supporting information




**Supplementary Figure**: brb371110‐sup‐0001‐Figure.docx

**Supplementary Table**: brb371110‐sup‐0002‐Table.xlsx

## Data Availability

The datasets analyzed in the current study were obtained from the following public resources: gene expression profiles (GSE49036, GSE20292, GSE157783, and GSE178265) were downloaded from the Gene Expression Omnibus (GEO; https://www.ncbi.nlm.nih.gov/geo); pQTL data were sourced from the UK Biobank (https://www.ukbiobank.ac.uk/) and the deCODE database (https://www.decode.com/); and PD phenotype data were accessed from the GWAS database (OpenGWAS ID: ieu‐b‐7; https://opengwas.io/). All data generated within this study are available within the article and its supplementary materials. Reasonable requests for additional information should be directed to the corresponding author.
