# Supplementary material for: CCNE1 Exerts a Protective Effect on Parkinson's Disease by Regulating Ferroptosis‐Related Proteins
Source: Brain Behav. 2025 Nov 29;15(12):e71110. doi: 10.1002/brb3.71110 (PMC12664902; doi:10.1002/brb3.71110)
Supplement: Supplementary file 1 — Supplementary Figure: brb371110‐sup‐0001‐Figure.docx [file BRB3-15-e71110-s001.docx]

**Supplementary Figure 1**


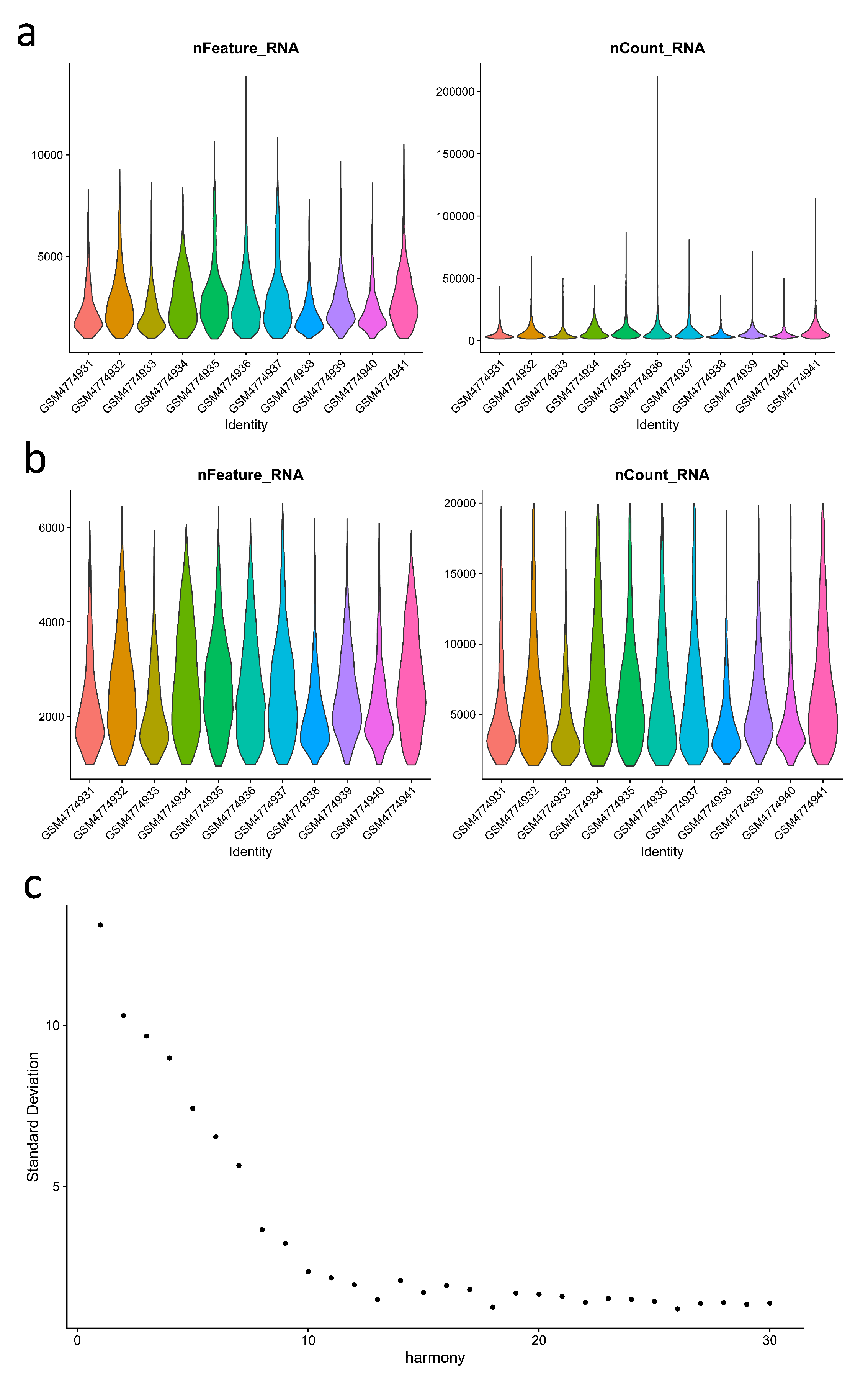


**Supplementary Figure 1.** Single-cell data analysis of GSE157783. (a) Distribution of nFeature_RNA, nCount_RNA before quality control, (b) Distribution of nFeature_RNA, nCount_RNA after quality control, (c) Fragmentation plots of PCs.

**Supplementary Figure 2**


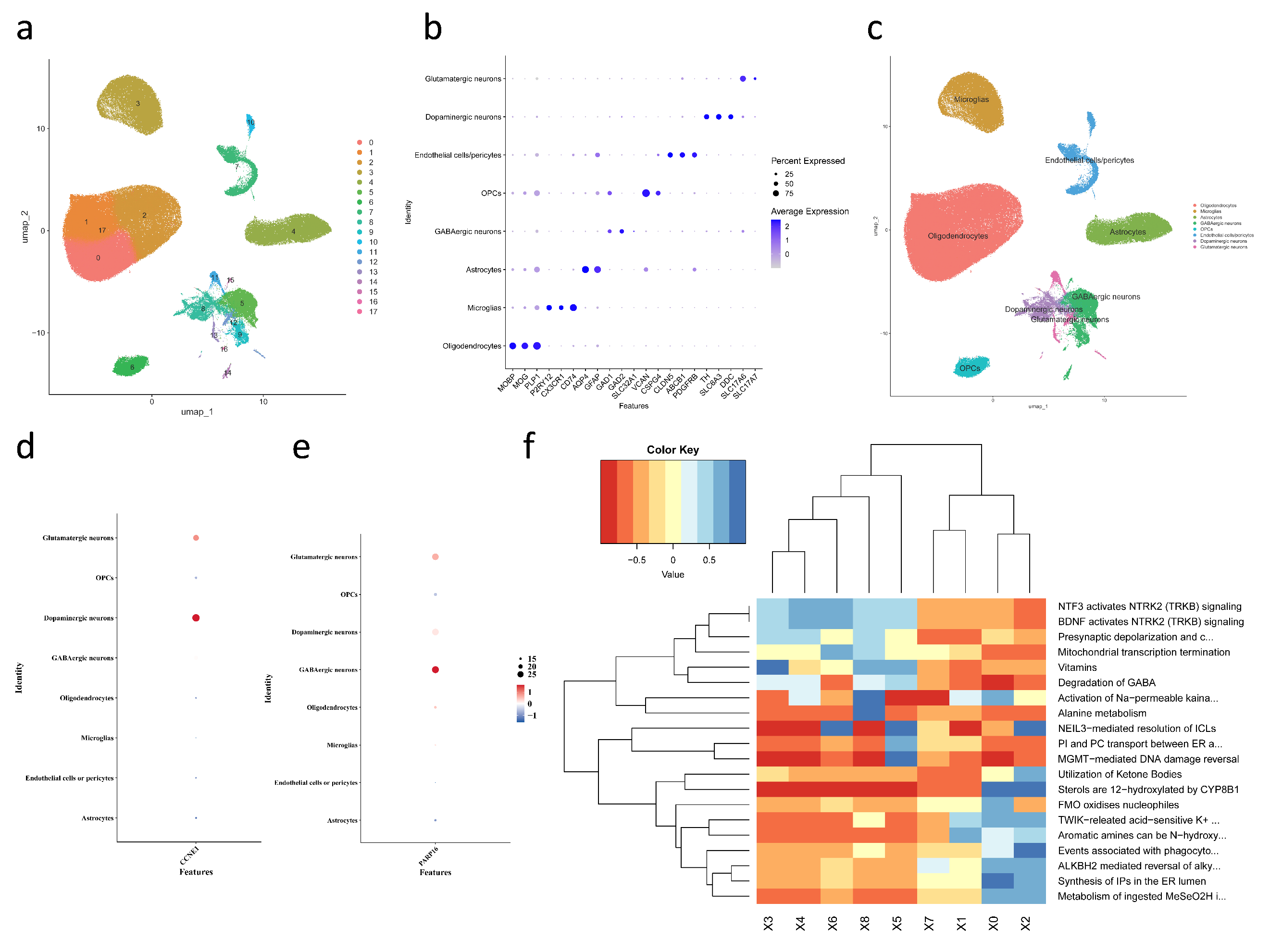


**Supplementary Figure 2.** Cellular clustering and expression profiles of key protein-coding genes in the human substantia nigra pars compacta (GSE178265). (a) UMAP visualization showing integrated cellular clustering. (b) Dot plot displaying expression patterns of canonical marker genes used for cell type annotation. (c) UMAP projection of the 18 identified cell clusters. (d) Expression distribution of CCNE1 across cell clusters visualized via dot plot. (e) Expression distribution of PARP16 across cell clusters visualized via dot plot. (f) Heatmap of functional enrichment terms specifically enriched in dopaminergic neurons.

**Supplementary Figure 3**


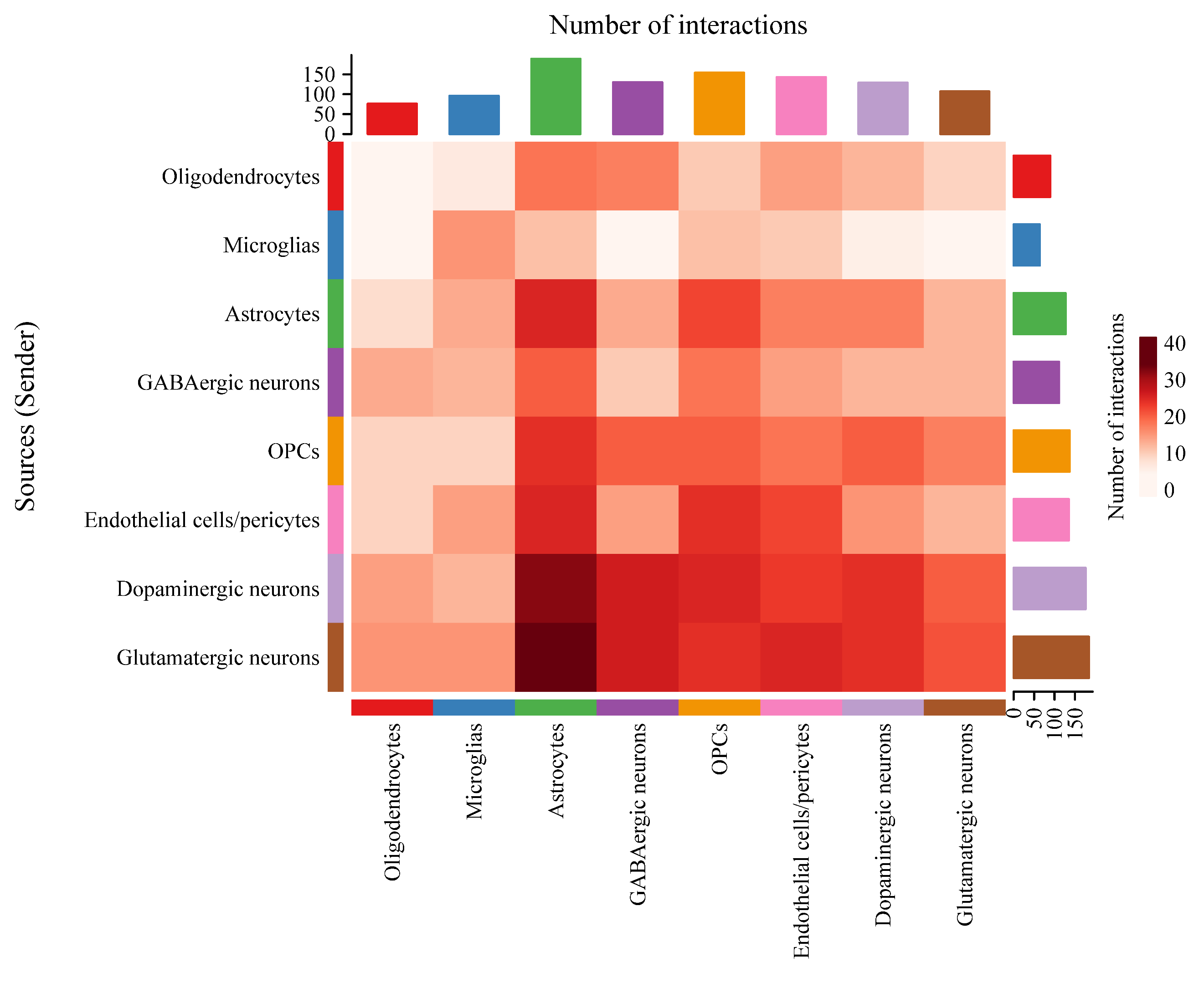


**Supplementary Figure 3.** Heatmap depicting the number of interactions among various cell types.

**Supplementary Figure 4**


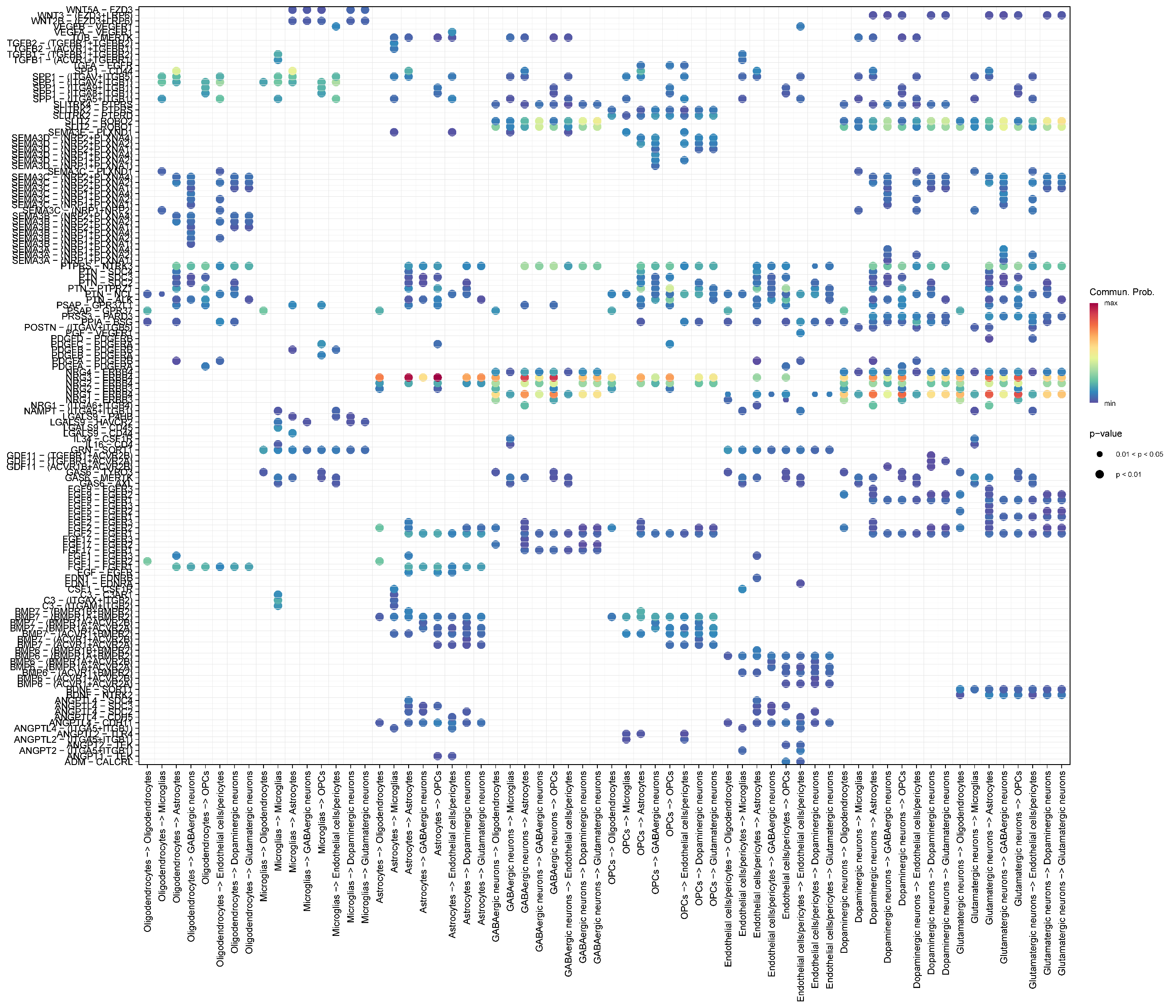


**Supplementary Figure 4.** Receptor-ligand interaction network visualized via proportional bubble plots.
